# Folic Acid and Vitamin B12 Administration in CKD, Why Not?

**DOI:** 10.3390/nu11020383

**Published:** 2019-02-13

**Authors:** Irene Capelli, Giuseppe Cianciolo, Lorenzo Gasperoni, Fulvia Zappulo, Francesco Tondolo, Maria Cappuccilli, Gaetano La Manna

**Affiliations:** Department of Experimental Diagnostic and Specialty Medicine (DIMES), Nephrology, Dialysis and Renal Transplant Unit, S. Orsola Hospital, University of Bologna, 40138 Bologna, Italy; irene.capelli@gmail.com (I.C.); giuseppe.cianciolo@aosp.bo.it (G.C.); lorenzo.gasperoni3@gmail.com (L.G.); fulvia.zappulo@studio.unibo.it (F.Z.); francesco.tondolo@studio.unibo.it (F.T.); maria.cappuccilli@unibo.it (M.C.)

**Keywords:** cardiovascular disease, chronic kidney disease, end-stage renal disease, hyperhomocysteinemia, folic acid, vitamin B12

## Abstract

Patients affected by chronic kidney disease (CKD) or end-stage renal disease (ESRD) experience a huge cardiovascular risk and cardiovascular events represent the leading causes of death. Since traditional risk factors cannot fully explain such increased cardiovascular risk, interest in non-traditional risk factors, such as hyperhomocysteinemia and folic acid and vitamin B12 metabolism impairment, is growing. Although elevated homocysteine blood levels are often seen in patients with CKD and ESRD, whether hyperhomocysteinemia represents a reliable cardiovascular and mortality risk marker or a therapeutic target in this population is still unclear. In addition, folic acid and vitamin B12 could not only be mere cofactors in the homocysteine metabolism; they may have a direct action in determining tissue damage and cardiovascular risk. The purpose of this review was to highlight homocysteine, folic acid and vitamin B12 metabolism impairment in CKD and ESRD and to summarize available evidences on hyperhomocysteinemia, folic acid and vitamin B12 as cardiovascular risk markers, therapeutic target and risk factors for CKD progression.

## 1. Introduction

Patients affected by chronic kidney disease (CKD) or end-stage renal disease (ESRD) have a shorter life expectancy than those with normal renal function, primarily due to the dramatic increase in cardiovascular mortality [1]. Chronic hemodialysis treatment is associated with a 10 to 50-fold higher risk of premature death than in the general population, and cardiovascular disease (CVD) represents the leading cause of death in hemodialysis patients [2,3]. Nevertheless, such increased cardiovascular risk is present since earlier stages of CKD [4].

In randomized clinical trials (RCTs), the traditional Framingham factors, such as hypertension, dyslipidemia and diabetes mellitus have been proven to be poor predictors of cardiovascular risk in this population. Therefore, there has been growing attention on non-traditional cardiovascular risk factors, in particular oxidative stress, endothelial dysfunction, chronic inflammation, vascular calcification Chronic Kidney Disease—Mineral and Bone Disorder (CKD-MBD) and hyperhomocysteinemia [5].

The “homocysteine hypothesis” arises from the observation that subjects with very high homocysteine blood levels due to congenital homocysteine metabolism impairment are more susceptible to develop a severe form of progressing atherosclerosis. Thus, over the years, research has been conducted into the possible link between an even moderate rise in homocysteine levels and cardiovascular risk and mortality, with conflicting results [6,7].

Although patients with CKD and ESRD display elevated homocysteine levels, the role of hyperhomocysteinemia as a cardiovascular and mortality risk factor in this population is still to be fully elucidated and deserves further investigation [8,9,10,11,12].

Furthermore, the high prevalence of hyperhomocysteinemia in patients with CKD has increased interest in speculating the role for hyperhomocysteinemia as a risk factor for the progression of CKD [13,14].

The role of folic acid and vitamin B12 role is well recognized, as they are not only essential cofactors for homocysteine metabolism, but their homeostasis disruption may be related directly to cardiovascular risk and CKD progression [11,15].

The aim of this review was to summarize folic acid, vitamin B12 and homocysteine metabolism in CKD patients and to analyze the published evidences on folic acid and vitamin B12 deficiency as cardiovascular risk markers and therapeutic targets in CKD and ESRD patients.

## 2. B Vitamins—Homocysteine Pathway

B vitamins, including vitamin B9 (folate) and vitamin B12 (cobalamin) are water-soluble vitamins involved in several normal cellular functions: they are providers of carbon residues for purine and pyrimidine synthesis, nucleoprotein synthesis and maintenance in erythropoiesis [16].

Folic acid is derived from polyglutamates that are converted into monoglutamates in the bowel, and then transported across mucosal epithelia by a specific carrier. The circulating form of folic acid is 5-methyltetrahydrofolate (5-MTHF) [17].

Vitamin B12, ingested with nutrients such as cobalamin, complexes with salivary haptocorrin, and is released abruptly from cobalamin by pancreatic proteases in the duodenum. Then, cobalamin, binds to an intrinsic factor secreted from the parietal cells of the stomach: when this complex arrives at the distal ileum, it is endocytosed from the enterocytes through cubilin. Then, cobalamin is carried into the plasma by a plasma transport protein named transcobalamin [16]. B12 is filtered by the glomerulus; however urine excretion is minimal due to reabsorption in the proximal tubule.

In target tissues, cobalamin is metabolized into two active forms: adenosylcobalamin in the mitochondria and methylcobalamin in the cytosol. Methylcobalamin is a methyl-transferring cofactor to the enzyme methionine synthase allowing homocysteine remethylation to methionine [17].

Homocysteine is a thiol-containing amino acid, not involved in protein synthesis, deriving from methionine metabolism. Plasma levels of homocysteine depend on several factors, such as genetic alteration of methionine metabolism enzymes or deficiency of vitamin B12, vitamin B6 or folic acid [18].

Methionine is transformed into S-adenosylmethionine (SAM) and then converted in S-adenosylhomocysteine (SAH) through a reaction catalyzed by methionine synthase reductase (MTRR). SAM, one of the most important methyl group donors, is formed within mitochondria and is a cofactor for a mutase known as methylmalonyl-CoA-mutase. This enzyme converts methylmalonyl-CoA into succinyl CoA, representing a crucial step in the catabolism of various amino acids and fatty acids. These processes also require pyridoxine (vitamin B6) as a cofactor [18].

**Figure 1:**
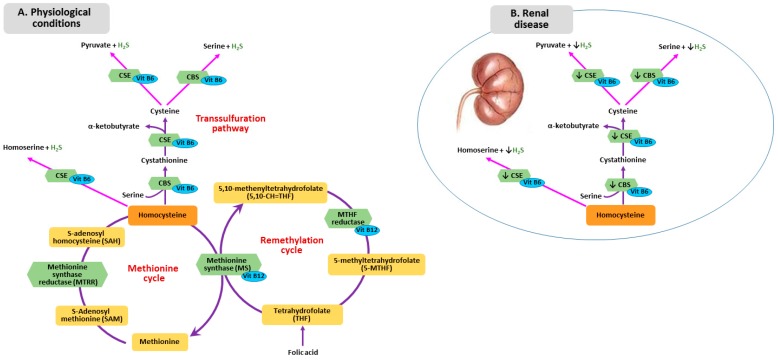
Homocysteine Metabolism in physiological condition (**A**) and in renal disease (**B**). CSE: cystathionine gamma-lyase; CBS: cystathionine beta synthase.

Homocysteine is the final product derived from hydrolysis of SAH to homocysteine and adenosine. Metabolism of homocysteine includes two different pathways: remethylation and transsulfuration (Figure 1A). In the remethylation pathway, methionine is regenerated through a reaction catalyzed by the enzyme methionine synthase (MTS), requiring folate and vitamin B12 as cofactors. Given that folate is not biologically active, it necessitates transformation into tetrahydrofolate that is then converted into methylenetetrahydrofolate (MTHF) by the enzyme methylenetetrahydrofolate reductase (MTHFR) [19].

The other pathway responsible for the homocysteine metabolism is transsulfuration. First, homocysteine combines with serine forming cystathionine by cystathionine beta synthase (CBS), then, cystathionine is hydrolyzed into cysteine and α-ketobutyrate by cystathionine γ-lyase (CTH) Human CBS is expressed in the liver, kidneys, muscle brain and ovary, and during early embryogenesis in the neural and cardiac systems [20].

The sulfur atom, in the form of sulfane sulfur or hydrogen sulfide (H_2_S), can be involved in vitamin B12-dependent methyl group transfer [21,22]. Alterations in methylation pathway, which causes a reduction of proteins and DNA methylation, results in abnormal vascular smooth muscle cell proliferation and increased lipid peroxidation [23]. Sulfur is a side product of conversion of homocysteine to cysteine by the enzymes CBS and cystathionine gamma-lyase (CSE). H_2_S is an angiogenic agent with antioxidant and vasorelaxing properties. Moreover, H_2_S represents an endogenous gaseous mediator, similarly to nitric oxide (NO) and carbon monoxide [24], which plays a role in several physiological processes, namely vascular smooth muscle relaxation, inhibition of vascular smooth muscle cell proliferation and blood pressure lowering [25]. Li et al. proved that H_2_S metabolism impairment might contribute to the development of uremia-associated accelerated atherosclerosis in CKD patients with diabetic nephropathy [26]. Patients with CKD and ESRD show lower H_2_S plasma levels, which can result from downregulation of CBS and CSE, mediated by hyperhomocysteinemia (Figure 1B). Whether this phenomenon can be attributed to additional factors is still unclear [21].

Homocysteine can be found in reduced and oxidized form in the bloodstream: more than 90% of the total plasma homocysteine is oxidized and bound to proteins, while the remaining oxidized homocysteine exists as a disulfide form. Only 2% of the total homocysteine in plasma is present as a free reduced form [27].

Normal homocysteine plasmatic level is <10 mmol/L, concentrations >10; however, levels <16 mmol/L are defined as mild hyperhomocysteinemia, while severe hyperhomocysteinemia is diagnosed when homocysteine >100 mmol/L [28].

Homocysteine is minimally eliminated by the kidney, since in physiological conditions, only non-protein bound homocysteine is subjected to glomerular filtration, and then for most part reabsorbed in the tubuli and oxidized to carbon dioxide and sulfate in the kidney cells [25].

Moreover, in the kidney, homocysteine is above all transsulfurated and deficiency of this renal transsulfuration contributes to the elevation of plasma homocysteine [18].

## 3. Metabolism of Homocysteine, Folic Acid and Vitamin B12 in CKD

Patients with CKD and ESRD have been shown to have higher homocysteine blood levels compared to the general population [8,29]. It has been hypothesized that hyperhomocysteinemia in these patients may be induced by the abnormality of homocysteine metabolism in the kidneys rather than by reduced glomerular filtration rate. In fact, although free homocysteine can pass the ultrafiltration barrier due to its low molecular weight, it circulates in the bloodstream mostly (about 90%) in the protein-bound form [27]. In particular, transsulfuration and remethylation pathways occurring in the kidney may be affected by renal disease. Stable isotope studies in nondiabetic and diabetic patients with CKD have shown impaired metabolic clearance of homocysteine determined by dysfunction in both pathways [30]. 

In both CKD and ESRD patients, several metabolic alterations, including acidosis, systemic inflammation and hormonal dysregulation, together with comorbidities and multidrug therapies, can lead to malnutrition with subsequent folic acid and vitamin B12 deficiency. In addition, anorexia, gastroparesis, slow intestinal transit or diarrhea, increased gut mucosal permeability and gut microbiota impairment may represent worsening factors [31,32]. 

Folic acid metabolism is impaired in uremic patients. Organic and inorganic anions, whose clearance is reduced in CKD, inhibit the membrane transport of 5-MTHF, thus compromising the incorporation into nucleic acids and proteins. Data suggest that transport of folates is slower in uremia and this implicated that, even with normal plasmatic folate levels, the uptake rate of folates into tissues may be altered [33]. In fact, serum folate concentration does not represent a reliable measure of tissue folate stores, but rather reflects recent dietary intake of the vitamin. Erythrocyte folate concentration is a better indicator of whole folate status. In a population of 112 dialysis patients, Bamonti et al. found serum folate levels normal in only 37% of cases, despite over 80% of red blood cells folate levels within the normal range [34]. 

Regarding vitamin B12, several studies have shown a correlation between low serum vitamin B12 concentrations and high BMI, insulin resistance, type 2 diabetes, dyslipidemia and CVD [35]. Vitamin B12 in the blood is primarily protein-bound. Approximately 20% of circulating B12 is bound to transcobalamin: this is the biologically active form that can be taken up into cells. Although CKD patients display increased transcobalamin levels, they show an impaired vitamin tissue uptake of B12 [36]. Moreover, in uremic patients a functional vitamin B12 deficiency can be observed because of increased transcobalamin losses in the urine and reduced absorption in the proximal tubule. This can lead to a “paradoxical” increase in cellular homocysteine levels despite normal total B12 [37].

On the other hand, potentially overdosage-related vitamin B12 toxicity could result exacerbated in individuals with CKD. Cyanocobalamin, the most commonly used form of B12 supplementation therapy, is indeed metabolized to active methylcobalamin, releasing small amounts of cyanide whose clearance is reduced in CKD [34]. Under normal conditions, methylcobalamin is required to remove cyanide from the circulation through conversion to cyanocobalamin. However, in CKD patients, the reduced cyanide clearance prevents conversion of cyanocobalamin to the active form and therefore supplementation is less effective [38]. 

The appropriate range of B12 levels in CKD remains to be defined adequately. Downstream metabolites, such as methylmalonic acid and homocysteine, may more accurately reflect functional B12 status in uremic patients [35].

## 4. Homocysteine-Mediated Tissue Damage

The pathogenic role of hyperhomocysteinemia on cardiovascular system in CKD and ESRD is related to atherosclerosis progression in the context of an already enhanced risk of vascular damage determined by uremic syndrome. One possible mechanism is the induction of local oxidative stress, generating Reactive Oxygen Species (ROS) because of the thiol group, which rapidly undergoes autoxidation in the presence of oxygen and metal ion. Besides, hyperhomocysteinemia promotes Nicotinamide Adenine Dinucleotide Phosphate (NADPH) oxidase activity with further increase in ROS generation. Hyperhomocysteinemia also determines Nitric Oxide (NO) metabolism impairment in endothelial cells (including Nitric Oxide Synthase expression, localization, activation, and activity) leading, together with ROS-induced local microinflammation, to endothelial dysfunction [39].

In cultured endothelial cells, hyperhomocysteinemia has been shown to upregulate monocyte chemotactic protein 1 (MCP-1) and interleukin-8 (IL-8) production, resulting in monocyte adhesion to the endothelium [40]. The link between homocysteine and inflammatory factors seems to be the activated transcription factor NF-κB (nuclear factor kappa-light-chain-enhancer of activated B cells) [41].

Additionally, hyperhomocysteinemia induces vascular smooth muscle cells (VSMC) proliferation by promoting the expression of adhesion molecules, chemokine and VSMC mitogen leading to several interactions with platelets, clotting factors and lipids [42], and might contribute to the scavenger receptor-mediated uptake of oxidized- Low Density Lipoprotein (LDL) by macrophages, triggering foam cell formation in atherosclerotic plaque [43,44,45,46]. Hyperhomocysteinemia also determines a vascular remodeling process that involves activation of metalloproteinase and induction of collagen synthesis, with subsequent reduction of vascular elasticity [47]. 

Likewise, elevated blood levels of homocysteine can cause endothelial reticulum stress with increase endothelial apoptosis and inflammation through a process mediated by ROS production and NF-κB activation [48,49,50]. Endothelial cells are known to be particularly vulnerable to hyperhomocysteinemia, since they do not express CBS, the first enzyme of the hepatic reverse transsulfuration pathway, or betaine-homocysteine methyltransferase (BHMT), which catalyzes the alternate remethylation pathway in the liver using betaine as a substrate [51].

Lastly, N-homocysteinylation of proteins is one process responsible for homocysteine toxicity, since it causes structural and functional loss. For LDL, homocysteinylation produces aggregation, accumulation of cholesterol and formation of foam-cells. Fibronectin is also involved in N-homocysteinylation: this reaction contributes to extracellular matrix remodeling, promoting the development of sclerotic processes [52]. 

Some peculiar effects of hyperhomocysteinemia on renal tissue have been described. Homocysteine can act directly on glomerular cells inducing sclerosis, and it can initiate renal injury by reducing plasma and tissue level of adenosine. Decreased plasma adenosine leads to enhanced proliferation of VSMC, accelerating sclerotic process in arteries and glomeruli. In a rat model of hyperhomocysteinemia induced by a folate-free diet, glomerular sclerosis, mesangial expansion, podocyte dysfunction and fibrosis occurred due to enhanced local oxidative stress. After treatment of the animals with apocynin, a NADPH oxidase inhibitor, glomerular injury was significantly attenuated [53].

## 5. Folic Acid and Vitamin B12 Impairment and Tissue Injury

Both folic acid and vitamin B12 have shown a potential direct relationship with cardiovascular outcomes with mechanism unrelated to homocysteine levels, although not clearly understood [54].

Folic acid improves endothelial function without lowering homocysteine, suggesting an alternative explanation for its effect on endothelial function that is possibly related to its anti-inflammatory, anti-oxidative and anti-apoptotic properties [55,56,57]. Experimental models revealed that folic acid can reduce endothelial dysfunction through the limitation of oxidative stress generation and the increasing of NO half-life [17]. 5-MTHF, the circulating form of folic acid, acutely improves NO-mediated endothelial function and decreases superoxide production. Moreover, 5-MTHF prevents oxidation of BH4 increasing enzymatic coupling of eNOS, enhancing NO production. Because 5-MTHF is a reduced form of folic acid that does not require conversion by dihydrofolate reductase, some direct effects may be attributable to redox mechanisms that are not seen when oral folic acid is used to increase plasma folate levels [58,59].

Doshi et al. investigated the direct effects of folic acid on endothelial function in patients with coronary artery disease (CAD) through Flow Mediated Dilatation (FMD) measurement before and after folic acid intake. FMD improved at 2 h in parallel with folic acid blood concentration, while homocysteine blood level did not change significantly. These data suggest that folic acid improves endothelial function in CAD acutely by a mechanism largely independent of homocysteine [60]. Other authors demonstrated that high-dose folic acid (5 mg/day) improves endothelial function in CAD patients with an action not related to homocysteine level [60,61,62,63]. We have previously reported that supplementation with 5-MTHF versus folic acid improved survival rate without differences in homocysteine levels [11]. Pan et al. recently showed that folic acid treatment can inhibit atherosclerosis progression through the reduction of VSMC dedifferentiation in high-fat-fed LDL receptor-deficient mice [64].

From the vitamin B12 side, patients with chronic inflammation, such as the hemodialysis population, display decreased production of transcobalamin II, due to impaired uptake of circulating B12 by peripheral tissues. This can determine increased synthesis of transcobalamins I and III that brings to further accumulation of B12 in blood [65,66,67,68]. Therefore, in the context of inflammatory syndromes, despite high vitamin B12 blood levels, there is a vitamin B12 deficiency in target tissues, potentially leading to hyperhomocysteinemia and increased cardiovascular risk [69].

Concerning anemia, unless CKD and ESRD patients show significant folate depletion, additional supplementation of folic acid does not appear to have a beneficial effect on erythropoiesis or on responsiveness to Recombinant Human Erythropoietin (rHuEPO) therapy. However, a diagnosis of folate deficiency should be considered in such patients when significant elevation in mean cell volume or hypersegmented polymorphonuclear leucocytes are found, especially in subjects with malnutrition, history of alcohol abuse, or in patients hyporesponsive to rHuEPO. Measurements of circulating serum folate do not necessarily mirror tissue folate stores, and red blood cell folate measures provide a more accurate picture. Low red blood cells folate concentrations in these patients suggest the need for folate supplementation [70].

In patients with CKD, folate and vitamin B12 deficiency may represent an influential factor in renal anemia and hyporesponsiveness to rHuEPO therapy. As such, the possibility and the requirement of a regular supplementation is still a matter of debate [71]. 

**Figure 2:**
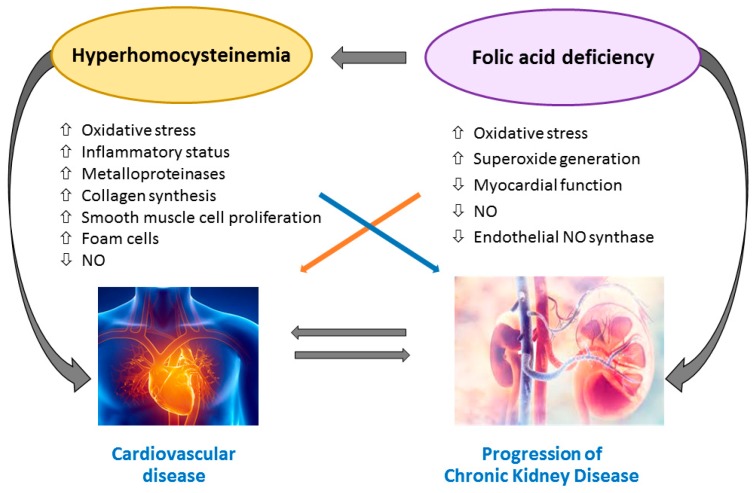
Hyperhomocysteinemia-induced amplification of atherosclerosis and inflammation in chronic kidney disease (CKD) patients. Abbreviations: NO, Nitric Oxide.

Figure 2 illustrates the pathways involved in the amplification of atherosclerosis and inflammation triggered by hyperhomocysteinemia in CKD patients.

## 6. MTHFR Gene Polymorphisms

MTHFR is an enzyme that plays a fundamental role in folate and homocysteine metabolism by catalyzing the conversion of 5,10-methenyltetrahydrofolate into 5-MTHF, the main circulating form of folate [72]. Several *MTHFR* gene polymorphisms have been described, and some of them seem to affect the individual susceptibility to a number of pathological conditions associated with homocysteine disorders, like myocardial infarction, stroke, neurodegenerative diseases, autoimmune diseases, cancer, diabetes, birth defects and kidney disease [73]. 

The most characterized are four functional single nucleotide polymorphisms at position 677 (*MTHFR* 677 C > T), at position 1298 (*MTHFR* 1298 A > C), at position 1317 (*MTHFR* 1317 T > C) and at position 1793 (*MTHFR* 1793 G > A) [74].

Although some studies excluded an association between *MTHFR* 677 C > T genotype and long-term kidney outcomes [75], *MTHFR* 677 C > T polymorphism has been shown to contribute to increase cardiovascular risk in ESRD patients [76]. A study of 2015 by Trovato et al. on 630 Italian Caucasian subjects found a lower frequency of *MTHFR* 677 C > T and A1298 A > C polymorphisms among ESRD patients requiring hemodialysis, suggesting a protective role of these gene variants on renal function [77]. 

Despite the fact that the main function of the MTHFR enzyme is to regulate the availability of 5-MTHF for homocysteine remethylation, the pathological consequences of functional variants of *MTHFR* gene cannot only be attributed to the increase in homocysteine levels. While the homocysteine lowering effect of routine folate supplementation in general population has been proven, patients with ESRD seem to display a folate resistance even to higher doses of folate [78]. Folate and vitamin B12 supplementation effects on hemodialysis patients are controversial, and possibly dependent on *MTHFR* polymorphisms [79]. 

Anchour et al. recently evaluated folic acid response in terms of homocysteine lowering with respect to *MTHFR* polymorphism carrier status in a prospective cohort of 132 hemodialysis patients. The authors found that 677 C > T *MTHFR* genotype influences vitamin B supplementation response, as reported in previous studies [79,80,81,82,83,84,85,86]. In particular, simultaneous supplementation of vitamin B12 and folate was useful only for the homozygous for the C allele, and the homocysteine reduction was significantly higher in carriers of TT genotype than in other genotypes [84].

Other authors reported that after B12 supplementation, homocysteine reduction in CC carriers was higher than in CT or TT carriers [82]. A renal substudy of the China Stroke Primary Prevention Trial (CSPPT) evaluated the effects of the combination of Angiotensin Converting Enzyme (ACE) inhibitors and folic acid with ACE inhibitors alone in reducing the risk of renal function decline in a hypertensive population without folic acid fortification. In 7545 patients treated with 10 mg enalapril and 0.8 mg folic acid, out of 15,104 participants, the greatest drop in serum homocysteine was in TT homozygotes of *MTHFR* 677 C > T polymorphism compared to other genotypes (CC/CT) [87].

In summary, the majority of available evidences suggest that *MTHFR* polymorphisms may influence folic acid and vitamin B12 treatment response in terms of homocysteine lowering and cardiovascular risk reduction in patients with CKD and ESRD on dialysis although indication of routine testing is matter of debate [88].

## 7. Role of Folic Acid, Vitamin B12 and Homocysteine as Cardiovascular Risk Markers

Although hyperhomocysteinemia has been accepted for years as a cardiovascular risk factor, its association with CVD and mortality has been recently questioned and literature data are controversial [7,89,90]. Epidemiologic and case-control studies generally support an association of elevated plasma homocysteine levels with an increased incidence of CVD and stroke, whereas prospective, randomized, placebo-controlled studies do not [7].

Moreover, a discrepancy still exists about the indication of routine screening for hyperhomocysteinemia and its treatment in the general population [7].

For CKD and ESRD patients, in spite of the increased homocysteine levels (average homocysteine level in the general population about 10–15 mmol/L versus 25–35 mmol/L in uremic patients), the role of homocysteine as a cardiovascular and mortality risk factor is still uncertain and many retrospective and interventional studies resulted in conflicting evidences [8,9,10,11,12].

A meta-analysis including retrospective studies, prospective observational studies and interventional trials (total population 5123 patients) showed that elevated homocysteine blood levels represent a risk factor for both CVD and mortality in patients with ESRD not treated with folic acid supplementation [10].

The prospective studies included in the meta-analysis showed that in unsupplemented patients with ESRD, an increase of 5 mmol/L in homocysteine concentration is associated with an increase of 7% in the risk of total mortality and an increase of 9% in the risk of cardiovascular events [10]. Conversely, in a prospective cohort of 341 hemodialysis patients, we previously failed to demonstrate a relationship between baseline homocysteine as well as *MTHFR* polymorphisms and mortality [11].

At the origin of these divergences, several possible factors may be hypothesized, such as non-homogeneous populations selection, temporal discrepancies between competitive risk factors and influence of common complication including inflammation and protein-energy wasting (PEW) that could influence circulating homocysteine and that are associated with poorer outcomes [9].

An inverse correlation between homocysteine levels and cardiovascular outcomes in advanced CKD and in hemodialysis patients has also been documented, configuring the phenomenon known as “reverse epidemiology” that also involves other cardiovascular risk factors, including Body Mass Index (BMI), serum cholesterol and blood pressure [91]. Some evidence indeed suggests that the presence of PEW and inflammation may justify the observed reverse association between homocysteine and clinical outcome in CKD and ESRD patients [34,35,36,37]. Specifically, two studies showed that patients with very low homocysteine plasma levels had worse outcomes, as confirmed by a higher incidence of hospitalization and mortality [92,93].

These data call into question the reliability of homocysteine as a marker of cardiovascular risk and mortality in patients with CKD and ESRD, raising the suspicion that other mechanisms beyond elevated homocysteine levels might be implicated. Given that DNA methyltransferases are among the main targets of hyperhomocysteinemia, it has been hypothesized that epigenetic alterations could play a role in hyperhomocysteinemia-mediated tissue damage [12].

Sohoo et al. recently carried out a retrospective study on a large cohort of hemodialysis patients investigating the association between baseline folic acid and vitamin B12 levels and all-cause mortality after an observation period of 5 years (9517 patients for folic acid group and 12,968 patients for B12 group). The authors found that higher B12 concentrations (550 pg/mL) were associated with a higher risk of mortality after adjusting for sociodemographic and laboratory variables, while only lower serum folate concentrations (<6.2 ng/mL) were associated with a higher risk of all-cause mortality. The authors pointed out that additional adjustment for malnutrition, inflammation and other clinical and laboratory variables nullified the folate–mortality association [15].

In our previous report, we demonstrated an improvement in survival rate of hemodialysis patients treated with 5-MTHF compared to folic acid, despite no difference in homocysteine levels between the two groups of treatment, raising the question whether 5-MTHF may have unique properties, unrelated to homocysteine lowering. Our finding of elevated CRP levels association with mortality allowed us to hypothesize that such effect may be mediated by a reduction in inflammation [11]. This raises the questions whether any benefit can be gained from lowering homocysteine and what role homocysteine actually plays in contributing towards cardiovascular events. (Table 1)

## 8. Effect of Folic Acid and Vitamin B12 Supplementation on CVD and Mortality in CKD and ESRD

Regarding folic acid and vitamin B12 supplementation, the role such vitamins administration with the aim of reducing mortality and prevent progression to ESRD is still to be determined. 

Moreover, effective folic acid and vitamin B12 supplementation dosages are not clearly established in the category of patients that take dosages ranging from 2.5 to 5 mg of folic acid three times a week up to more than 15 mg/day. Simultaneous administration of intravenous B complex vitamins is proven to be more efficient in reducing homocysteine serum levels and restoring the remethylation pathway in ESRD patients [116]. 

Righetti et al. in a one-year, placebo-controlled, non-blinded randomized control trial on a cohort of 81 chronic hemodialysis patients, showed no survival benefit of treatment with folic acid compared to placebo, and only 12% of patients on treatment reached normal homocysteine blood levels [117]. Wrone et al. found no difference in terms of mortality and cardiovascular events in a multicentre study on 510 patients on chronic dialysis randomized to 1, 5, or 15 mg/day of folic acid [76].

In the ASFAST study (Cardiovascular Morbidity and Mortality in the Atherosclerosis and Folic Acid Supplementation Trial), a double blinded, placebo controlled trail, a randomized cohort of 315 CKD dialysis patients (with eGFR < 25 mL/min) were treated with folic acid 15 mg/die or placebo. After a median follow-up of 3.6 years, the results failed to demonstrate a benefit of folic acid therapy regarding all-cause mortality, cardiovascular mortality and control of atheroma progression (carotid intima-media thickness progression) [118].

The HOST trial (Homocysteinemia in Kidney and End Stage Renal Disease) is a double blind, placebo-controlled trial in which 2056 patients with advanced CKD or ESRD requiring renal replacement therapy and elevated homocysteine levels, were randomized to a combined therapy with folic acid, vitamin B12 and piridoxin or placebo. After a median follow-up of 3.2 years, the study showed a significant reduction in homocysteine levels, but failed to reach its primary end-point, reduction of all-cause mortality, and its secondary end-point, reduction in cardiovascular death, amputation and thrombosis of the vascular access. A possible explanation for these negative results may be ascribed to the high cardiovascular comorbidity burden and the suboptimal compliance to therapy. Moreover, the study considered CKD and ESRD population together and was underpowered to evaluate the two populations separately. The disparity between these findings and the previously reported epidemiologic data could reflect limitations of observational studies [119].

Recently, Heinz et al. designed a multicenter trial on 650 chronic hemodialysis patients randomized to 5 mg folic acid, 50 µg vitamin B12 and 20 mg vitamin B6 versus placebo three times a week (post-dialysis) for 2 years. No differences were observed between the two groups in terms of all cause-mortality and fatal and non-fatal cardiovascular events. On the other side, post-hoc analysis revealed a significant reduction in unstable angina pectoris and fewer vascularization procedures [120].

In a meta-analysis by Heinz et al. involving five intervention trials for a total of 1642 dialysis patients treated with folic acid, vitamin B12 and vitamin B6, a significant CVD risk reduction but not mortality risk reduction was demonstrated [10]. Another meta-analysis including 3886 patients with ESRD or advanced CKD (creatinine clearance < 30 mL/min) assessed the relationship between folic acid therapy (with or without vitamin B6 and B12) and CVD. Folic acid reduced cardiovascular risk by 15% in ESRD patients with greater benefit in those treated for longer than 24 months and in those from areas with no or partial grain fortification [121].

Ji et al. performed a large meta-analysis including 14 RCTs (54,913 participants) that demonstrated overall stroke events reduction resulting from homocysteine lowering following folic acid, vitamin B12 and vitamin B6 supplementation. Beneficial effects in reducing stroke events were observed in the subgroup with CKD [122]. A meta-analysis of 10 studies concluded that homocysteine-lowering therapy is not associated with a significant decrease in the risks for CVD events, stroke, and all-cause mortality among patients with CKD. It has to be pointed out that, respect to previous meta-analysis a high number of participants with diabetes were included and that RCTs were performed in grain fortification areas [123]. More recently, a meta-analysis took into account six studies (comprising the abovementioned ones) for 2452 patients on chronic hemodialysis, finding no significant differences in mortality and in the incidence of cardiovascular events in patients treated with homocysteine lowering therapy [124].

Finally, many published post-hoc analyses have shown that several factors including age, baseline homocysteine levels, folic acid fortification of grains, B12 status, renal function, comorbidities and medications could be modifiers of folic acid and vitamin B12 effects on cardiovascular risk [12]. 

Table 2 summarizes the interventional studies investigating the effects of folic acid and vitamin B12 administration on CVD risk, mortality and CKD progression. 

To our knowledge, there are no published prospective studies that specifically addressed the effect of vitamin B12 alone on cardiovascular or renal outcomes in CKD and ESRD patients.

In summary, although the available trials indicate a reduction in homocysteine levels with medical therapy (folic acid, vitamin B12 and vitamin B6), in the majority of cases a benefit on mortality and on the incidence of cardiovascular events in patients with CKD and ESRD has not been demonstrated. Moreover, the beneficial effect could depend on anti-inflammatory and vascular protective effects. 

## 9. Folic Acid and Vitamin B12: Evidences on CKD Progression

Regarding the relationship between folic acid, vitamin B12 supplementation and CKD progression, available interventional studies have demonstrated no clear benefit or even harmful effects on renal outcomes [15], while observational studies showed a correlation between hyperhomocysteinemia and risk of CKD development and progression [129].

The China Stroke Primary Prevention Trial (CSPPT) is a large RCT (20,702 patients) that enrolled adults with hypertension without a history of stroke or myocardial infarction with determination of *MTHFR* genotype and baseline folate level. The study aimed at evaluating the effect of treatment with folic acid in an Asian population without folic acid fortification. Authors found that the ACE inhibitors plus folic acid therapy, compared with ACE inhibitors alone, reduced stroke risk. Moreover, subjects with the CC or CT *MTHFR* genotype had the highest risk of stroke and the greatest benefit of folic acid supplementation while those with the TT genotype required a higher dosage of folic acid to reach sufficient levels [80].

A renal sub-study of the China Stroke Primary Prevention Trial (CSPPT) compared the efficacy of combination of enalapril and folic acid with enalapril alone in reducing the risk of renal function decline in a large hypertensive population (15,104 patients). The study included patients with eGFR greater than 30 mL/min and participants were randomized to receive enalapril 10 mg plus folic acid 0.8 mg or enalapril 10 mg alone. Compared with the enalapril group, the enalapril-folic acid group showed reduced risk of CKD progression by 21% and a reduction of eGFR decline rate of 10% after a four-year follow-up. Authors performed a subgroup analysis that compared patients with CKD (defined as eGFR < 60 mL/min or presence of proteinuria) and without CKD at baseline. It resulted that CKD at baseline was a strong modifier of the treatment effect [85]. Specifically, the greatest decrease in serum homocysteine was in TT homozygotes of *MTHFR* 677 C > T polymorphism, while the magnitude of the declines in those with CC/CT genotypes was smaller. Finally, an exploratory subgroup analysis aimed to assess the treatment effect on the primary outcome in various subgroups among CKD participants showed that CKD progression risk reduction was more represented in diabetes subgroup [87]. This has been the first study showing renal protection from folic acid therapy in a population without folic acid fortification. Previous trials have reported a null or harmful effect of supplementation with folic acid and vitamin B12 [15,118]. 

In the abovementioned HOST study, treatment with high doses of folic acid, vitamin B6 and vitamin B12 failed to delay the time to initiating dialysis in patients with advanced CKD [15].

Diabetic Intervention with Vitamins to Improve Nephropathy (DIVINe) was a double blind RCT on a population of 238 patients with diabetic kidney disease randomized to receive 2.5 mg folic acid plus 25 mg vitamin B6 plus 1 mg vitamin B12, or placebo (mean eGFR was 64 mL/min in treatment group and 58 mL/min in the placebo group). Results showed that treatment with folic acid, vitamin B6 and vitamin B12 led to a greater decrease in GFR and to an increase in cardiovascular events compared to placebo after a follow-up of 2.6 years [125]. The possible explanations for such conflicting results may be multiple. First of all, baseline folic acid levels were different between studies (7.7 ng/mL for CSPPT renal sub study, 15 and 16.5 ng/mL for DIVINe and HOST study respectively) corroborating the hypothesis that beneficial effects of folic acid supplementation on renal outcomes could be stronger in patients with low folic acid level at baseline. In addition, vitamin B doses may play a role, as suggested by the elevated folate blood levels reached in the HOST study (2000 ng/mL) compared to CSPPT (23 ng/mL). This allowed postulating a potential toxicity determined by unmetabolized folic acid accumulated in the bloodstream [85]. Finally, CKD severity differed between the studies. In fact, the HOST study population was composed by patients with advanced CKD and with high comorbidity burden and this could have attenuated the study power with respect to renal outcomes. The fact that the CSPPT renal sub study demonstrated a benefit of folic acid therapy on the progression of renal damage could be related to the choice of a population with mild-moderate CKD. Furthermore, only CSPPT trial selected a population without folic acid grain fortification, while the other two studies were carried out in countries with folic acid fortification programs.

Besides, a recent study on 630 Italian Caucasian population found a lower frequency of *MTHFR* 677 C > T and A1298 A > C polymorphisms among dialysis patients compared to subjects without or with slight-moderate renal impairment, suggesting a protective role of both polymorphisms on renal function [39].

In conclusion, the available evidences regarding the effect of homocysteine lowering therapies on CKD progression are controversial and further studies with CKD progression as primary end-point and more homogeneous population selection are needed. While awaiting further evidences, it seems reasonable to treat patients with folic acid deficiency in order to reduce the risk of CKD progression avoiding accumulation phenomena that could lead to toxicity.

## 10. Folic Acid and Vitamin B12 in Kidney Transplant Recipients

In kidney transplant recipients several factors such as dialytic history, anemia, immunosuppression, inflammatory state and dysmetabolic alterations may influence cardiovascular risk [130].

A decline in homocysteine blood levels after kidney transplantation is frequently observed; nonetheless, hyperhomocysteinemia usually persists [131,132,133]. It has been documented that homocysteine can be further lowered among stable transplant recipients through high-dose B-vitamin therapy [134]. The effect of folic acid, vitamin B12 and vitamin B6 supplementation on cardiovascular risk and mortality reduction has been investigated by the Folic Acid for Vascular Outcome Reduction in Transplantation (FAVORIT) trial. Stable transplant recipients were randomized to daily multi-vitamin drug containing high-doses of folate (5.0 mg), vitamin B12 (1.0 mg) and vitamin B6 (50 mg) or placebo. The study was terminated early after an interim analysis because, despite effectively homocysteine lowering action, the incidence of CVD, all-cause mortality and onset of dialysis-dependent kidney failure did not differ between the treatment arms [135].

A longitudinal ancillary study of the FAVORIT trial recently showed that high-dose B-vitamin supplementation determined modest cognitive benefit in patients with elevated baseline. It has to be pointed out that almost all subjects had no folate or B12 deficiency; thus, the potential cognitive benefits of folate and B12 supplementation in individuals with poor B-vitamin status remains controversial [136].

## 11. Conclusions

At present, the available evidence does not provide full support to consider hyperhomocysteinemia, folic acid and vitamin B12 alterations reliable cardiovascular disease and cardiovascular mortality risk markers in CKD and ESRD populations. Furthermore, such factors do not represent a validated therapeutic target regarding the reduction of cardiovascular risk and CKD progression.

While waiting for the results of confirmatory trials, it seems reasonable to consider folic acid with or without vitamin B12 supplementation as appropriate adjunctive therapy in patients with CKD.

Concerning patients in early CKD stages for which potassium or phosphorus dietary intake restriction is not indicated, folic acid could come in the form of a healthy diet rich in natural sources of folate. For patients with advanced CKD and on dialysis, folic acid can be supplemented pharmacologically after accurate folate status assessment.

## Figures and Tables

**Table 1:** Retrospective and prospective observational studies on hyperhomocysteinemia and folic acid/vitamin B12 impairment in patients with CKD and end-stage renal disease (ESRD).

Study	Design	Participants, n	Case Definition/Outcome	Results
Soohoo et al., 2017 [15]	Retrospective	9517 (folate group), 12968 (B12 group) HD	Mortality	Lower folic acid predicts mortality
Ye et al., 2016 [94]	Cross-sectional	1042 CKD stage 1–5	CVD	HHcy associated with CKD severity, LVH, LVD and vascular disease
Anan et al., 2006 [95]	Retrospective case-control	44 HD	Silent cerebral infarction	HHcy predicted outcome
Ducloux et al., 2006 [92]	Prospective observational	459 HD	Mortality and fatal CVD	HHcy predicted outcome only in patients without CIMS
Nair et al., 2005 [96]	Retrospective case-control	146 HD	MI, heart surgery	HHcy did not predict CVD risk
London et al., 2004 [97]	Prospective observational	78 HD	Mortality	HHcy did not predict outcome
Kalantar-Zadeh et al., [98]	Prospective observational	367 HD	Mortality	Lower Hcy levels predicted mortality
Buccianti et al., 2004 [99]	Prospective observational	77 HD	Fatal CVD	HHcy predicted outcome
Bayès et al., 2003 [100]	Prospective observational	94 HD	Mortality, fatal CVD	HHcy did not predict outcome
Mallamaci et al., 2002 [101]	Prospective observational	175 HD	Mortality, fatal CVD	HHcy predicted outcome
Ducloux et al., 2002 [102]	Prospective observational	240 PD	Fatal and nonfatal CVD	HHcy did not predict outcome
Haraki et al.,2001 (retrospective part) [103]	Retrospective case-control	43 HD/PD	Coronary, cerebral and peripheral vascular disease	HHcy CVD risk factor
Haraki et al., 2001 (prospective part) [103]	Prospective observational	55 HD/PD	Fatal and nonfatal CVD	HHcy predicted outcome
Wrone et al., 2001 [104]	Retrospective case-control	459 HD/PD	MI, stroke, TIA, carotid endarterectomy.	HHcy did not predict CVD risk
Dierkes et al., 2000 [105]	Prospective observational	102 HD	Mortality, fatal/nonfatal CVD	HHcy predicted outcome
Suliman et al., 2000 [106]	Retrospective case-control	117 HD	Coronary, cerebral and peripheral vascular disease	HHcy did not predict CVD risk
Kunz et al., 1999 [107]	Retrospective case-control	63 HD	Coronary, cerebral and peripheral vascular disease	HHcy cardiovascular risk factor
Manns et al., 1999 [108]	Retrospective case-control	218 HD	Coronary, cerebral and peripheral vascular disease	HHcy cardiovascular risk factor only in males
Sirrs et al., 1999 [109]	Prospective observational	88 HD	Mortality and CVD events	Lower Hcy levels predicted mortality
Moustapha et al., 1998 [110]	Prospective observational	167 HD/PD	Mortality and CVD events	HHcy predicted outcome
Vychytil et al., 1998 [111]	Retrospective case-control	154 PD	Coronary, cerebral and peripheral vascular disease	HHcy did not predict CVD risk
Bostom et al., 1997 [112]	Prospective observational	73 HD/PD	CVD events	HHcy predicted outcome
Robinson et al., 1996 [113]	Retrospective case-control	176 HD/PD	Coronary, cerebral and peripheral vascular disease	HHcy cardiovascular risk factor
Bachmann et al., 1995 [114]	Retrospective case-control	45 HD	Coronary, cerebral and peripheral vascular disease	HHcy cardiovascular risk factor
Bostom et al., 1995 [115]	Retrospective case-control	24 HD/PD	Coronary, cerebral and peripheral vascular disease	HHcy did not predict CVD risk

Abbreviations: CVD, Cardiovascular Disease; MI, Myocardial Infarction; LVH, left ventricular hypertrophy; LVD, left ventricular dysfunction; HHcy, hyperhomocysteinemia; HD, Hemodialysis; PD, peritoneal dialysis; CKD, Chronic Kidney Disease; CIMS, chronic inflammation-malnutrition state.

**Table 2:** Interventional trials on the effects of folic acid and vitamin B12 administration and CVD risk, mortality and CKD progression.

Study	Design/Intervention	Participants, *n*	End Point	Follow-up, Years	Results
Xu et al., 2016 [87]	Double blind RCT: enalapril 10 mg versus enalapril 10 mg plus folic acid	15,104 (eGFR ≥ 30 mL/min). No folic acid fortification	CKD progression	4.4	Enalapril plus folic acid delayed CKD progression
House et al., 2010 [125]	Double blind RCT: folic acid 2.5 mg + Vitamin B6 25 mg + Vitamin B12 1 mg versus placebo	238 (diabetic nephropathy with eGFR > 30 mL/min). Folic acid fortification	CKD progression	2.6	Greater GFR decrease and more CVD events in treatment group
Heinz et al., 2010 [120]	Double blind RCT: folic acid 5 mg, vitamin B12 50 µg, vitamin B6 20 mg versus placebo 3 times a week	650 hemodialysis patients	All-cause mortality, cardiovascular events	2	No differences
Mann et al., 2008 [126]	Double blind RCT: folic acid 2.5 mg + vitamin B6 50 mg + vitamin B12 1 mg versus placebo	619 CKD (eGFR <60 mL/min)	All-cause mortality, cardiovascular events	5	No differences
Cianciolo et al., 2008 [11]	Open label randomized trial: 5-MTHF intravenous. three times a week versus folic acid 5 mg oral daily	314 hemodialysis patients	All-cause mortality	4.5	Less mortality risk in 5-MTHF group (independent of homocysteine)
Jamison et al., 2007 [119]	Double blind RCT (HOST): folic acid 40 mg + vitamin B6 100 mg + vitamin B12 2 mg versus placebo	2056 CKD (eGFR ≤ 30) or hemodialysis (folic acid fortification)	All-cause mortality, CKD progression	3.2	No differences
Vianna et al., 2007 [127]	Double blind RCT: folic acid 5 mg versus placebo	97 hemodialysis patients	Cardiovascular events	2	No differences
Zoungas et al., 2006 [118]	Double blind RCT (ASFAST): folic acid 15 mg versus placebo	315 CKD (eGFR < 25 mL/min), hemodialysis and peritoneal dialysis	Cardiovascular events and mortality	3.6	No differences
Righetti et al., 2006 [128]	Open prospective trial: folic acid 5 mg versus untreated	114 hemodialysis patients	Cardiovascular events	2.4	Folic acid decreases CVD events
Wrone et al., 2004 [76]	Three arms, double blind RCT: folic acid 1 mg or 5 mg or 15 mg	510 hemodialysis patients	Cardiovascular events and mortality	2	No differences
Righetti et al., 2003 [117]	Placebo-controlled, non-blinded RCT: folic acid 5, 15, 25 mg or placebo	81 hemodialysis patients	Cardiovascular mortality	1	No differences

Abbreviations: CKD, Chronic Kidney Disease; CVD, Cardiovascular Disease; eGFR, estimated Glomerular Filtration Rate; RCT, Randomized Clinical Trial.
